# Structures of EccB_1_ and EccD_1_ from the core complex of the mycobacterial ESX-1 type VII secretion system

**DOI:** 10.1186/s12900-016-0056-6

**Published:** 2016-02-27

**Authors:** Jonathan M. Wagner, Sum Chan, Timothy J. Evans, Sara Kahng, Jennifer Kim, Mark A. Arbing, David Eisenberg, Konstantin V. Korotkov

**Affiliations:** Department of Molecular & Cellular Biochemistry and Center for Structural Biology, University of Kentucky, 741 South Limestone, Lexington, KY 40536 USA; UCLA-DOE Institute, University of California Los Angeles, Los Angeles, CA 90095-1570 USA; UCLA-DOE Institute, Departments of Biological Chemistry and Chemistry & Biochemistry, and Howard Hughes Medical Institute, University of California Los Angeles, Los Angeles, CA 90095-1570 USA; Present address: Department of Molecular Physiology and Biological Physics and The Myles H. Thaler Center for AIDS and Human Retrovirus Research, University of Virginia, Charlottesville, VA USA; Present address: Division of Regulatory Services, College of Agriculture, Food and Environment, University of Kentucky, Lexington, KY USA

**Keywords:** EccB, EccD, *Mycobacterium tuberculosis*, Type VII secretion system, ESX

## Abstract

**Background:**

The ESX-1 type VII secretion system is an important determinant of virulence in pathogenic mycobacteria, including *Mycobacterium tuberculosis*. This complicated molecular machine secretes folded proteins through the mycobacterial cell envelope to subvert the host immune response. Despite its important role in disease very little is known about the molecular architecture of the ESX-1 secretion system.

**Results:**

This study characterizes the structures of the soluble domains of two conserved core ESX-1 components – EccB_1_ and EccD_1_. The periplasmic domain of EccB_1_ consists of 4 repeat domains and a central domain, which together form a quasi 2-fold symmetrical structure. The repeat domains of EccB_1_ are structurally similar to a known peptidoglycan binding protein suggesting a role in anchoring the ESX-1 system within the periplasmic space. The cytoplasmic domain of EccD_1_has a ubiquitin-like fold and forms a dimer with a negatively charged groove.

**Conclusions:**

These structures represent a major step towards resolving the molecular architecture of the entire ESX-1 assembly and may contribute to ESX-1 targeted tuberculosis intervention strategies.

**Electronic supplementary material:**

The online version of this article (doi:10.1186/s12900-016-0056-6) contains supplementary material, which is available to authorized users.

## Background

Pathogenic bacteria rely on a variety of secretion systems to transport virulence factors, proteins that mediate host-pathogen interactions, across their hydrophobic cell membranes to sites where they can interact with the host. Gram-positive bacteria need only transport proteins across a single membrane, but Gram-negative bacteria require specialized secretion machinery that spans both inner and outer membranes. *Mycobacterium tuberculosis*, the causative agent of tuberculosis, was recently re-classified as a diderm bacterium when it was shown to have an outer membrane bi-layer — referred to as the mycomembrane — composed largely of mycolic acids [1]. In order to transport key virulence factors across both membranes *M. tuberculosis* has evolved specialized Type VII secretion systems (T7SS). The T7SSs were discovered based on attenuated strains of *M. tuberculosis* deficient in EsxA (ESAT-6, early secreted antigenic target of 6 kDa) secretion and are commonly called **E**SAT **six** (ESX) secretion systems [2–4]. In *M. tuberculosis* there are five gene clusters, named ESX-1 to ESX-5, which encode T7SS. Each gene cluster encodes a number of proteins that are either secreted or are building blocks for the secretion apparatus. ESX-1 is responsible for secretion of the important virulence factors EsxA and EsxB as well as other virulence-associated proteins (e.g., EspB, EspF, EspJ) that are secreted to the cell surface or extracellular milieu based on recognition of a conserved C-terminal signal sequence on the secretion substrates [5–8]. These secreted factors have been linked to mycobacterial virulence through studies of the attenuated BCG strain of *M. tuberculosis* [2, 4, 9]; in non-pathogenic *Mycobacterium smegmatis* the orthologous ESX-1 system is involved in conjugation [10, 11]. ESX-3 is critical for mycobacterial survival due to its role in metal acquisition [12–14]. ESX-5 is important for the secretion of many members of the PE/PPE family of proteins that also play a role in virulence and cell wall integrity [15–17]. The functional role of ESX-2 and ESX-4 is still unknown although ESX-4 appears to be the ancestral system from which the other ESX systems have evolved [18].

All ESX gene clusters contain at least three or four ESX conserved components (Ecc), named EccB, EccC, and EccD, with EccE being present in all ESX systems with the exception of ESX-4 [19]. Multiple copies of each core protein as well as other T7SS-associated proteins are present in the core complex resulting in a large ~1500 kDa particle [20]. The function of some core components is known, for example, EccC is a member of the FtsK/SpoIIIE-like ATPase family and provides the energy to transport proteins across the mycobacterial membrane(s) [21, 22]. EccD contains an N-terminal cytoplasmic domain followed by 11 predicted transmembrane helices, and may form the cytoplasmic membrane channel through which cargo proteins are secreted. The functions of EccB and EccE within the secretion apparatus are less clear. These proteins both have N-terminal transmembrane elements and large C-terminal regions predicted bioinformatically to be localized in the periplasm, but their molecular structures and interacting partners remain unknown.

Understanding the T7SS architecture is critical for development of new antitubercular agents. Currently, no structural data is available for three of the four conserved components EccB, EccD, and EccE. In this study we report the molecular structures of the periplasmic domain of EccB_1_ and the cytoplasmic domain of EccD_1_ from the ESX-1 cluster. The structures reveal probable functional surfaces of EccB_1_, and an unexpected dimerization by EccD_1_. Here we describe these structures in detail and how they might fit into the larger context of the T7SS.

## Results and discussion

### Structure of EccB_1_

**Fig. 1:**
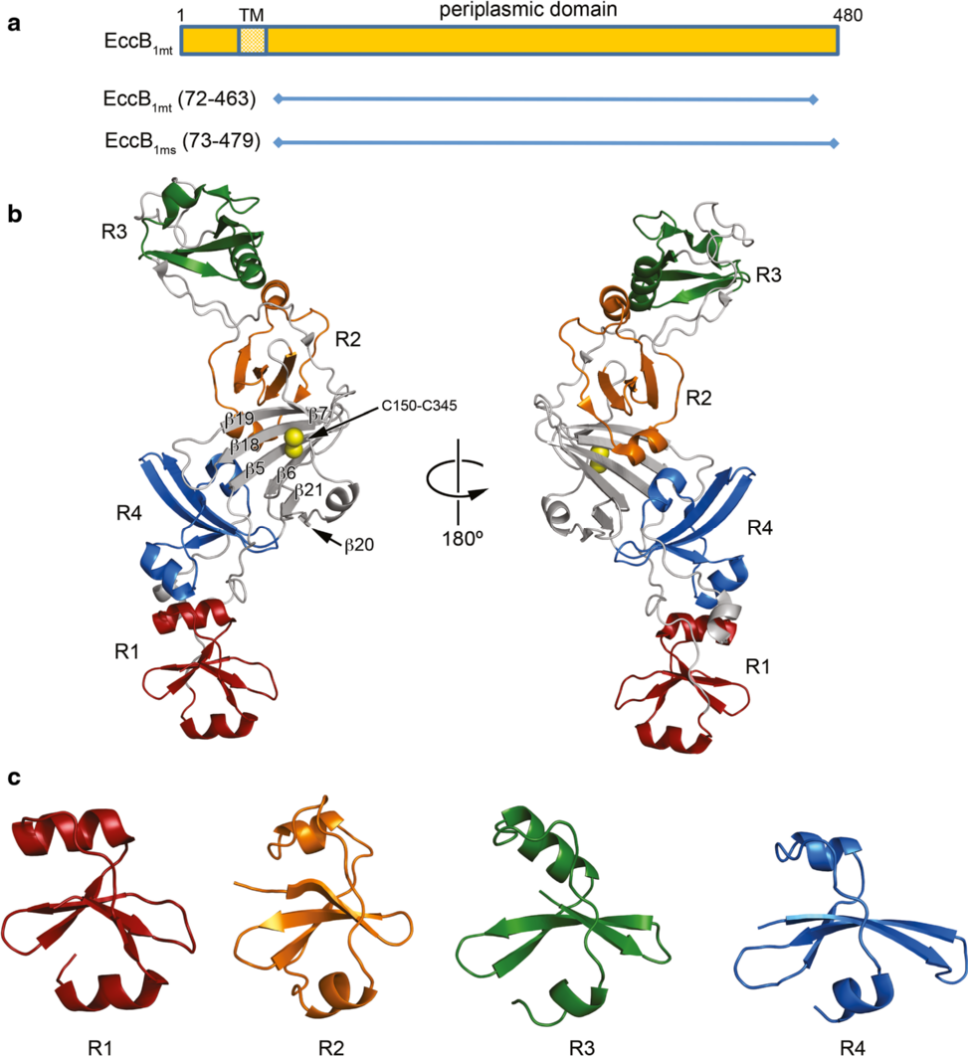
Overall structure and repeat domains of EccB_1mt_. **a** Domain organization of EccB_1_. The predicted transmembrane helix is indicated by a shaded rectangle. The protein variants used for structure determination are shown as horizontal lines. **b** Overall structure of EccB_1mt_. The structure is shown in cartoon representation with the central core domain in grey and repeats domains R1-R4 colored red, orange, green, and blue, respectively. The disulfide bond between Cys150 and Cys345 is shown as yellow spheres. **c** Repeat domains R1–R4 have a common fold. The isolated repeat domains are shown in the same orientation after superposition of repeats R2-R4 on repeat R1 using Chimera [52]

**Fig. 2:**
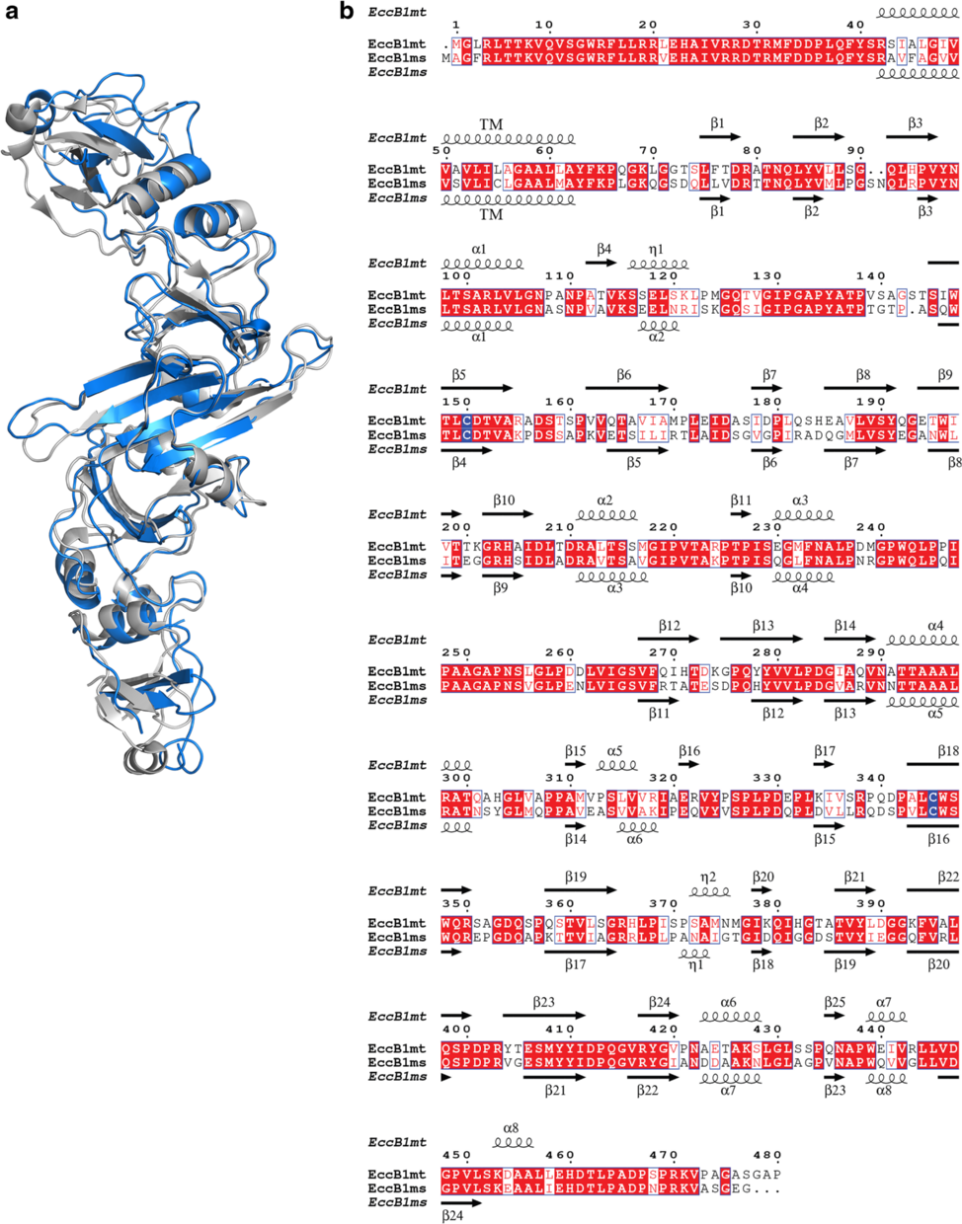
Superposition of EccB_1mt_ and EccB_1ms_ structures. **a** EccB_1mt_ (grey) and EccB_1ms_ (blue) were superimposed using Chimera. **b** Structure-based sequence alignment of EccB_1mt_ and EccB_1ms_ prepared with ESPript (http://espript.ibcp.fr) [53] with numbering and secondary structure elements derived from the EccB_1mt_ sequence and structure

*M. tuberculosis* EccB_1_ (Rv3869) is a 51 kDa protein containing a 40 amino acid (aa) N-terminal domain followed by a single membrane-spanning helix and a ~400 aa C-terminal fold. EccB_1_ is annotated as a protein domain of unknown function (DUF690) in the Pfam database [23]. In order to gain further insight into the role of EccB_1_ within the ESX machinery we determined the crystal structure of the C-terminal domains of EccB_1_ from *M. tuberculosis* (EccB_1mt_) to 1.7 Å resolution and of the orthologous protein (MSMEG_0060; EccB_1ms_) from the nonpathogenic mycobacterial species *M. smegmatis* to 3.07 Å resolution. Both EccB_1_ structures contain a single elongated fold in the shape of a distorted propeller, which has an unanticipated quasi 2-fold symmetry (Fig. 1). A structural comparison of the EccB_1mt_ and EccB_1ms_ structures shows that they are highly similar with an r.m.s.d. of 2.7 Å for the superposition of 381 amino acids (Dali Z-score 42.2); there is considerable variability in the conformation of the extensive unstructured loops connecting secondary structure elements which are, themselves, relatively well conserved (Fig. 2) Five domains are present in the structures including a core domain flanked by two repeat domains on either side. The central core domain consists of a 6 stranded β-sheet with 5 strands (β7-β19-β18-β5-β6) arranged in anti-parallel fashion with an additional strand (β21) parallel to strand β6 on the periphery of the sheet; the sheet is further stabilized by a disulfide bond between the two central strands (β5 and β18) of the sheet formed between Cys150 and Cys345 (EccB_1mt_) and Cys152 and Cys347 (EccB_1ms_). The four repeat domains each contain a 4 stranded β sheet and two α helices (Fig. 1c). Repeat 1 (R1) (residues S74-M124) and repeat 4 (R4) (residues G391-L445) are located between the core domain and the N-terminal transmembrane region while repeat 2 (R2) (residues E185-P241) and repeat 3 (R3) (residues V267-A320) are located on the opposite side of the core domain distal to the transmembrane region. The interfaces between R1/R4 and R2/R3 domains are formed by hydrophobic residues on the N-terminal helices of each repeat that fold together with each other and with hydrophobic residues from the proline rich strands downstream of each repeat’s C-terminal helix. The R2 and R3 domains also pack tightly with the core domain via residues on their N-terminal helices as well as β sheet residues. The tight packing involving residues on either side of multiple repeat domains gives EccB_1_ a stable fold with a continuous hydrophobic core and an elongated pseudo-symmetrical shape.

**Fig. 3:**
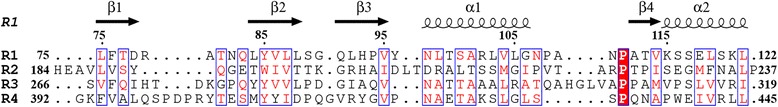
Structure-based sequence alignment of repeat domains of EccB_1mt_. Alignment was rendered using ESPript. Amino acid numbering above the alignment refers to the repeat domain R1 sequence and indicated secondary structure elements are derived from the repeat domain R1 structure

A comparison of the repeat domains of EccB_1mt_ gives clues to the evolution of the protein. Pairwise sequence alignments of the repeats shows that R2, R3, and R4 show 26, 33 and 27 % sequence identity, respectively, to R1. Pairwise alignments comparing R2–R4 to all other repeats revealed that only R1 has significant identity to all 3 other domains (Fig. 3). Therefore, it appears that R1 is the ancestral domain with R3 sharing more conserved features with R1 than do either R2 or R4. EccB_1ms_ contains a corresponding set of repeats in the same arrangement as seen in EccB_1mt_: R1 (residues Q75–K127) is membrane proximal, R4 (residues G392–L447), the central core domain, R2 (residues Q187–P243), and R3 (residues G267–E323) which is distal to the membrane.

EccB_1_ does not bear significant sequence similarity to any protein of known structure, and Dali searches using the complete EccB_1_ structures revealed no proteins with significant structural homology. However, Dali searches using only EccB_1mt_ repeat 1 (S74–P124) revealed weak homology (r.m.s.d. 2.7 Å and Dali Z-score of 5.0) to the N-terminal domain of PlyCB (PDB 4 F87, residues 14–70) from streptococcal C1 bacteriophage [24]. Eight PlyCB monomers assemble into a ring that associates with the bacterial cell wall and facilitate phage egress by tethering the degradative PlyCA subunit to the bacterial cell wall. The structural similarity between the two proteins and a common localization of both to bacterial cell envelope structures is intriguing but no clues to EccB_1_ function are apparent from our examination of PlyCB.

### Structure of EccD_1mt_

EccD_1mt_ (Rv3877) is a 54 kDa protein containing an ~110 amino acid (aa) N-terminal ubiquitin-like domain followed by a 30 aa linker and 11 closely spaced transmembrane helices at its C-terminus. The ubiquitin-like domain of EccD_1_ classifies it as a member of the YukD family within the Pfam database. Based on the characteristics of the transmembrane regions the N-terminal portion of EccD_1_ is predicted to be localized in the cytoplasm.

**Fig. 4:**
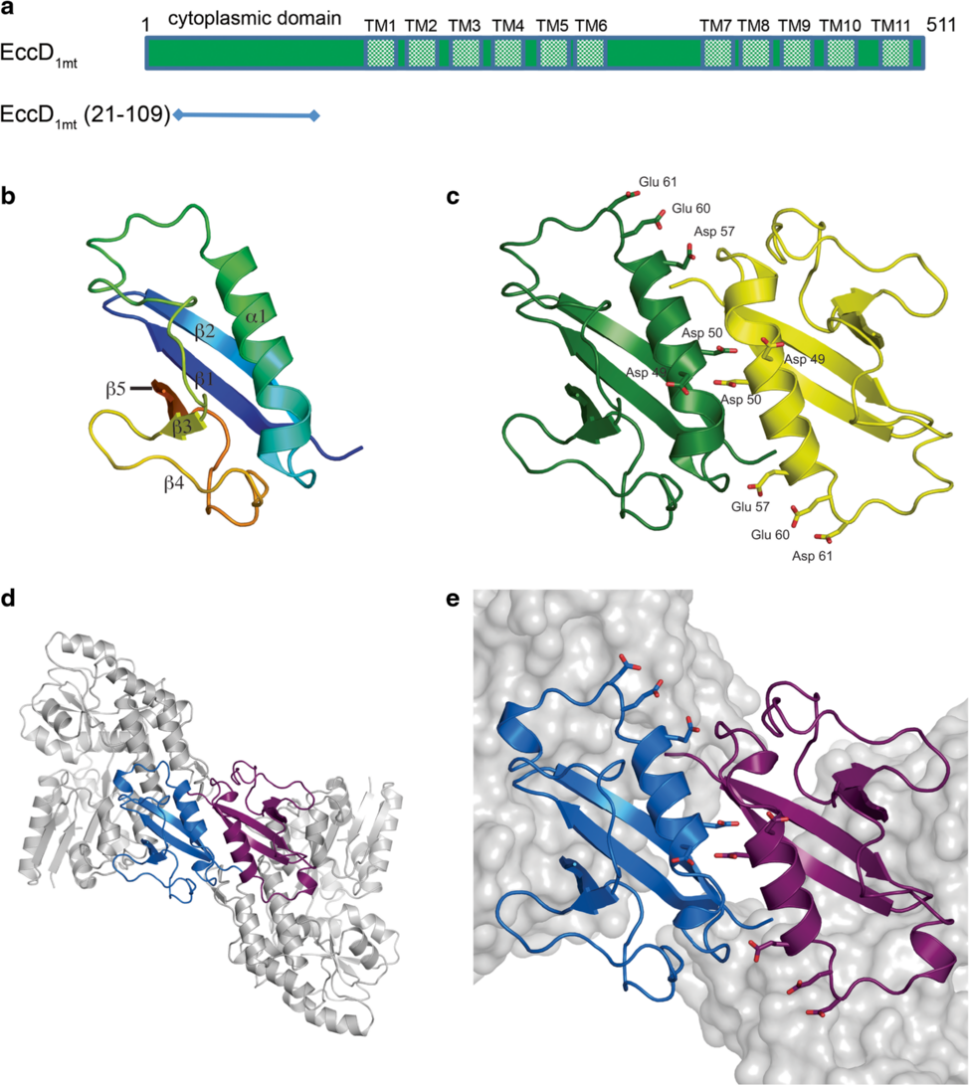
Structure of the cytoplasmic domain of EccD_1mt_. **a** Domain organization of EccD_1_. The predicted transmembrane helices 1–11 are indicated by shaded rectangles. The protein construct used for crystallization is shown as a horizontal line. **b** cyto-EccD_1mt_ monomer in cartoon representation colored in rainbow colors from N-terminus (blue) to C-terminus (red). The secondary structure elements are labeled. **c** cyto-EccD_1mt_ dimer in cartoon representation with acidic residues shown in stick representation (see Fig. 5). **d** MBP-cyto-EccD_1mt_ dimer in cartoon representation with MBP moieties colored in grey and cyto-EccD_1mt_ domains colored in blue and purple. **e** A close-up view of the MBP-cyto-EccD_1mt_ dimer. The orientation corresponds to panel **c**

We grew crystals of the predicted cytoplasmic domain of EccD_1_ from *M. tuberculosis* (cyto-EccD_1mt_) which diffracted to 1.88 Å. However, we could not obtain crystals of Se-Met containing cyto-EccD_1mt_ and attempts to perform heavy atom soaks of fragile native crystals of cyto-EccD_1mt_ were unsuccessful. Therefore, we obtained crystals and determined the structure of cyto-EccD_1mt_ fused to maltose binding protein (MBP) at a resolution of 2.20 Å by molecular replacement using an MBP structure (PDB ID 1ANF) as the search model [25]. We subsequently solved the 1.88 Å cyto-EccD_1mt_ structure by molecular replacement using the EccD_1mt_ segment of the MBP fusion protein. In both structures EccD_1mt_ residues 20–109 adopt an identical ubiquitin-like fold characterized by a β grasp motif and an anti-parallel β sheet with strands in the order 2,1,5,3,4 (Fig. 4). The MBP fusion protein used as a crystallization aid provides additional crystallization contacts, but it does not perturb the fold of cyto-EccD_1mt_ (Fig. 4d,e). The two EccD_1mt_ structures are superimposable with an r.m.s.d. of 0.7 Å over 90 residues and a Dali Z-score of 18.8.

Interestingly, the asymmetric unit of both crystal forms contains two EccD_1_ molecules and in both crystal forms the two EccD_1_ molecules are arranged as a head-to-tail homodimer stabilized by an extensive interface. The interface is formed by interlocking side chains from β strands 1 and 2 and the N-terminal α-helix of both EccD_1_ molecules (Fig. 4) and ~650 Å^2^ of each EccD_1_ molecule (13 % of the total surface) is buried in the interface as calculated with the PISA webserver [26]. The interaction is stabilized by 4 hydrogen bonds and a cluster of buried hydrophobic residues including Met1, Val54, and Val58 resulting in a solvation energy of −13.9 kcal/mol and a Complex Significance Score of 1.0 calculated by the PISA server. The extensive nature of the interface and its re-occurrence in both crystal forms, with or without the MBP fusion, suggests that EccD_1_ is a natural homodimer.

**Fig. 5:**
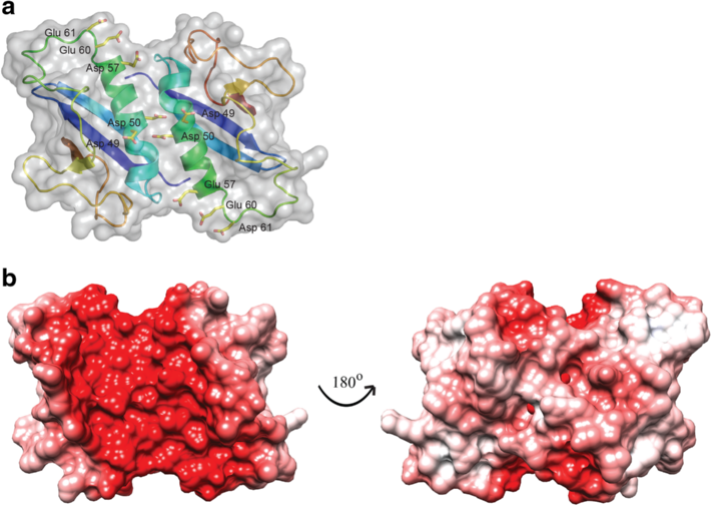
Dimerization of cyto-EccD_1mt_ creates a negatively charged groove. **a** cyto-EccD_1mt_ dimer is shown in cartoon representation underneath a semitransparent surface. Clustered acidic residues are shown in stick representation. **b** Electrostatic surface calculated using the APBS server [54] with protonation states at pH 7.0 assigned by PROPKA [55]. The surface was colored +10 eV (blue) to −10 eV (red)

Dimerization of cyto-EccD_1mt_ creates a wide open-ended groove bordered on two sides by the α1/β3 loops (Fig. 5). The floor of the groove is formed by the two α helices. Notably, the dimerization interface brings acidic residues (Glu45, Asp49, Asp50, Glu57, Glu60, and Asp61) from both chains into this groove. These acidic residues are not offset by the presence of any basic residues in this region thus they create a highly negative surface (Fig. 5b).

### Putative function of EccB_1_ and EccD_1_

Mutations in EccB_3_ of the ESX-3 secretion system have been shown to confer drug resistance in *M. tuberculosis* [27]. The mutations found to confer resistance (Arg14Leu, and Asn24His) occur in the small cytoplasmic domain preceding the transmembrane element of EccB_3_, a region not present in our EccB_1_ constructs which contain the soluble periplasmic domain. The fact that mutations in this region confer drug resistance indicates an important function for this short region perhaps in mediating interactions with other cytoplasmically exposed components of the T7SS. The elongated shape and continuous hydrophobic core of EccB_1_ suggest that it may serve a structural role – perhaps forming part of a structure that spans the inner and outer membrane components of the ESX secretion system. The structural similarities between PlyCB, the viral cell wall binding protein complex, and EccB_1_ hints that EccB_1_ may also bind elements of the peptidoglycan layer, but there is not yet any experimental data to support this idea. However, post-translational modification of secreted bacterial proteins with O-linked polysaccharides has been shown to be important for solubility or maintaining subcellular localization to the cell wall [28, 29]. EccB_1_ contains 24 putative glycosylation sites, as predicted by the NetOGlyc webserver [30], and many of these are surface-exposed in the EccB_1_ structures (including Ser143, Thr144, Ser351, and Ser356). While this manuscript was under preparation, the ATPase activity of EccB_1_ has been reported [31]. Further studies are needed to define the precise role of EccB_1_ in the context of a functional ESX-1 secretion complex.

The dimerization of the cytoplasmic domain of EccD_1_ raises interesting possibilities regarding the nature of the transmembrane pore. Each EccD_1_ monomer has 11 transmembrane elements thus a dimer would have a total of 22 transmembrane elements. Each monomer may form an independent pore resulting in a pair of closely associated channels, or the transmembrane elements may comprise a single, larger, transmembrane channel. The cytoplasmic domain itself is connected to the first transmembrane element by a 30 amino acid linker that may facilitate protein-protein interactions, either with the cytoplasmic EccD_1_ domain or other components of the secretion system, or it may simply form an extended tether allowing increased mobility of the ubiquitin-like domains.

The negatively charged groove of the EccD_1_ dimer indicates that it should associate with a positively charged partner(s). It may act to recruit other T7SS components or secretion substrates with positively charged patches into the system, or it may be part of a gating element required to close the channel during periods of inactivity. The residues contributing to the negatively charged groove are not conserved in EccD_1_ homologs from other ESX systems indicating that they may serve a system-specific role. Indeed, the ESX-1 locus encodes a variety of secretion substrates not found in the paralogous *M. tuberculosis* ESX systems and thus it is likely that the ESX-1 system has structural adaptations to enable the secretion of these substrates [6–8, 32]. As more structures of ESX-1 components are determined likely partners for interaction with the EccD_1_ dimer may be revealed.

## Conclusions

In summary, we have determined the structures of soluble domains of two integral, conserved components, EccB_1_ and EccD_1_, of the ESX-1 secretion channel. Given the importance of the ESX-1 secretion system to mycobacterial virulence, our structures provide crucial information about the molecular makeup of this important protein complex that will aid future drug development efforts.

## Methods

### Expression and purification of EccB_1mt_

A construct for expression of the periplasmic domain of EccB_1mt_ (residues 72–463) was designed based on predicted transmembrane helix using the TOPCONS server [33], secondary structure prediction using the JPred4 server [34], and the sequence alignment of EccB_1_ orthologs (Additional file 1: Figure S1). The DNA fragment was PCR-amplified from *M. tuberculosis* H37Rv genomic DNA using primers EccB1_F72_Nco 5′–CACCATGGGCACCAGCCTGTTCACCGACC and EccB1_RS463_Hind 5′–GCAAGCTTACAGCGTGTCGTGCTCGAGCAG, and cloned into a modified pET-22b(+) vector (Novagen), which contains the *Escherichia coli* DsbA signal sequence, a hexahistidine tag and a tobacco etch virus (TEV) protease cleavage sequence.

EccB_1mt_ was expressed in *E. coli* Rosetta2(DE3) strain using LB media and 0.5 mM IPTG for induction. Cells were harvested after 4 h incubation at 18 °C, resuspended in 20 mM Tris–HCl pH 8.0, 300 mM NaCl buffer, and lysed using microfluidizer (Avestin). EccB_1mt_ was purified via a Ni-NTA affinity column, incubated with TEV protease to remove the hexahistidine tag, and passed over a Ni-NTA column to remove uncleaved protein, and further purified by size exclusion chromatography using a Superdex 200 column (GE Healthcare). Protein was flash-frozen using liquid nitrogen and stored at −80 °C.

### Crystallization and structure determination of EccB_1mt_

Crystals were grown using the sitting drop vapor diffusion method with precipitant containing 0.1 M Tris–HCl pH 5.6, 15 % PEG2000 MME, 10 mM NiCl. Crystals were transferred to crystallization solution supplemented with 20 % glycerol, or with 20 % glycerol and 0.5 NaI [35], and flash-frozen in liquid nitrogen.

Data were collected at the 22-ID beamline at the Advance Photon Source, Argonne National Laboratory, and processed using *XDS* [36] and HKL-3000 [37]. Iodide ion positions were determined using SHELXD [38] as implemented in HKL-3000, and phases were calculated using SHARP [39]. The model was built using Buccaneer [40] and Coot [41], and refined by REFMAC5 [42] using TLS groups defined by the TLSMD server [43]. The final structure includes residues 74–458.

### Expression and purification of EccB_1ms_

The periplasmic domain (residues S73-G479) of the MSMEG_0060 gene was PCR-amplified from *M. smegmatis* mc^2^155 genomic DNA with the gene-specific primers MsEccB1.For. 5′-AACCTGTATTTCCAGAGTAGTGACCAGCTGCTGGTGG and MsEccB1.Rev. 5′-TTCGGGCTTTGTTAGCAGTTAGCCCTCCCCGCTCG and cloned into the pMAPLe4 expression vector [44], which appends a TEV protease cleavable hexahistidine tag to the N-terminus of the target protein, using the Gibson ISO cloning method [45]. The sequence of the expression clone was verified by DNA sequencing (Genewiz, Piscataway, NJ).

Recombinant protein was overexpressed in *E. coli* BL21(DE3) by inducing protein expression, of 1 L Terrific broth cultures, at an OD600 of 1.0 with the addition of IPTG to 0.5 mM. Cell growth was continued overnight at 18 °C. The following day the cells were harvested by centrifugation and resuspended in Buffer A (20 mM Tris, pH 8.0, 300 mM NaCl, 10 % Glycerol) containing 10 mM imidazole, 1 mM EDTA and Complete protease inhibitor and lysed by sonication. The lysate was clarified by centrifugation (15,000 × g, 30 min, 4 °C) and the supernatant was loaded on a Ni-NTA affinity column equilibrated in Buffer A. After extensive washing the bound protein was eluted with Buffer B (Buffer A containing 250 mM imidazole). The target protein was further purified by size exclusion chromatography using a Sephacryl S-100 column (GE Healthcare) equilibrated in Buffer A.

### Crystallization and structure determination of EccB_1ms_

Crystals of EccB_1ms_ were grown using the hanging drop vapor diffusion method by mixing protein at a 1:1 ratio of protein to reservoir solution (14 % PEG 8000, 200 mM NaCl, 100 mM phosphate-citrate pH 4.2). Crystals were cryoprotected by a brief soak in reservoir solution containing 20 % propylene glycol. Data from a single crystal was collected at beamline 24-ID-C at the Advanced Photon Source, Argonne National Laboratory. The data were processed with *XDS* [36] and the structure solved by molecular replacement using the program *Phaser* [46] and a homology model, prepared with the Phyre2 web server [47], based on the structure of *M. tuberculosis* EccB_1_ (PDB ID 4KK7). The structure was refined with BUSTER [48].

### Expression and purification of cyto-EccD_1mt_

A construct for expression of the cytoplasmic domain of EccD_1mt_ (residues 21–109) was designed based on predicted ubiquitin-like domain using the HHpred server [49]. The DNA fragment was PCR-amplified from *M. tuberculosis* H37Rv genomic DNA using primers EccD1_F21_Nco 5′–CACCATGGCCACCACCCGGGTGACGATC and EccD1_R109_SpeEcoR 5′–GGGAATTCACTAGTCATGACACCAGAGTCAGCAGTGAC, and cloned into a modified pET-Duet1 vector, which contains an N-terminal hexahistidine tag and TEV protease cleavage sequence. To create a maltose-binding protein (MBP) fusion construct, the same DNA fragment was cloned into a modified pET-22b(+) vector, which contains an N-terminal hexahistidine tag and TEV protease cleavage sequence followed by MBP sequence. Both cyto-EccD_1mt_ and MBP-cyto-EccD_1mt_ proteins were expressed and purified as described for EccB_1mt_. 5 mM maltose was included in the size-exclusion buffer during purification of MBP-cyto-EccD_1mt_ variant to obtain ligand-bound MBP [50].

### Crystallization and structure determination of MBP-cyto-EccD_1mt_ and cyto-EccD_1mt_

Crystals of cyto-EccD_1mt_ were obtained by sitting drop vapor diffusion method using 0.1 M Tris–HCl pH 8.5, 0.2 M Mg chloride, 30 % PEG4000 as precipitant. Crystals were cryoprotected using crystallization solution supplemented with 10 % glycerol, and vitrified in liquid nitrogen. Crystals grew as thin hexagonal plates and were mounted in cryo-loops with 60° tilt (Mitigen) to avoid overlapping reflections along the crystallographic *c* axis (Table 1). Crystals of MBP-cyto-EccD_1mt_ were obtained by sitting drop vapor diffusion method using 0.1 M HEPES pH 7.5, 1.4 M Na citrate, and cryoprotected using crystallization solution supplemented with 20 % glycerol.

**Table 1:** Diffraction data collection and refinement statistics

	EccB_1mt_ (PDB: 4KK7)	EccB_1ms_ (PDB: 5CYU)	cyto-EccD_1mt_ (PDB: 4KV2)	MBP-cyto-EccD_1mt_ (PDB: 4KV3)
Data collection				
Wavelength (Å)	1.0000	0.9789	1.0000	1.0000
Space group	*P*2_1_2_1_2_1_	*P*6_1_22	*P*6_5_22	*P*6_5_
Cell dimensions				
*a*, *b*, *c* (Å)	31.70, 110.63, 110.51	74.41, 74.41, 280.60	46.72, 46.72, 279.02	125.68, 125.68, 124.49
α, β, γ, (°)	90, 90, 90	90, 90, 120	90, 90, 120	90, 90, 120
Resolution (Å)	49.48–1.68 (1.77–1.68)^a^	64.44–3.07 (3.15–3.07)	46.50–1.88 (1.98–1.88)	49.91–2.20 (2.32–2.20)
*R*_sym_	0.053 (0.895)	0.103 (2.22)	0.106 (0.872)	0.105 (0.983)
CC_1/2_^b^	99.9 (50.0)	99.8 (56.0)	99.9 (88.1)	99.9 (82.9)
*I*/σ*I*	14.2 (1.4)	13.7 (1.0)	20.6 (3.5)	17.7 (3.2)
Completeness (%)	94.1 (88.8)	92.9 (91.2)	100.0 (100.0)	100.0 (100.0)
Multiplicity	4.7 (3.0)	8.3 (7.7)	13.4 (13.9)	11.5 (11.5)
Refinement				
Resolution (Å)	49.48–1.68	64.44–3.07	46.50–1.88	49.91–2.20
No. reflections (total/free)	42769/2118	8759/805	15842/794	56603/2862
*R*_work_/*R*_free_	0.177/0.214	0.241/0.297	0.188/0.236	0.163/0.205
No. atoms				
Protein	2870	2650	1332	6970
Ligand/ion	18	0	0	46
Water	347	10	186	418
*B*-factors				
Protein	29.2	92.3	28.4	42.2
Ligand/ion	30.2			26.3
Water	36.8	42.6	34.5	42.3
Wilson *B*	33.8	113.7	30.1	63.0
R.m.s. deviations				
Bond lengths (Å)	0.009	0.008	0.010	0.008
Bond angles (°)	1.347	1.060	1.331	1.220
Ramachandran distribution (%)^c^				
Favored	97.4	92.2	100.0	98.2
Outliers	0.0	0.0	0.0	0.2

^a^Values in parentheses are for the highest-resolution shell

^b^Half-set correlation coefficient CC_1/2_ as defined in Karplus and Diederichs [56] and calculated using *XSCALE* [36] or Scala [57]

^c^Calculated using the MolProbity server (http://molprobity.biochem.duke.edu) [58]

Data were collected at the 22-ID beamline at the Advance Photon Source, Argonne National Laboratory, and processed using *XDS* [36]. The structure of MBP-cyto-EccD_1mt_ was solved by molecular replacement using *Phaser* [46] and an MBP structure as a search model (PDB ID 1ANF) [25]. The electron density modification was performed using Parrot [51], and the model was extended using Buccaneer and Coot. The fragment corresponding to cyto-EccD_1mt_ from the structure of MBP-cyto-EccD_1mt_ was used as a search model to solve the structure of cyto-EccD_1mt_ alone using *Phaser*. The structures were refined using REFMAC5 and TLS groups defined by the TLSMD server.

## Availability of supporting data

The structure factors and atomic coordinates have been deposited in the Protein Data Bank under accession codes 4KK7 (EccB_1mt_), 5CYU (EccB_1ms_), 4KV2 (cyto-EccD_1mt_), and 4KV3 (MBP-cyto-EccD_1mt_).

## Additional file

Additional file 1: Figure S1. Sequence alignment of EccB orthologs from M. *tuberculosis* H37Rv. The secondary structure elements of EccB_1mt_ are shown at the top of the alignment. The conserved Cys residues are highlighted in blue. The vertical arrows indicate the beginning and end of the EccB_1mt_ expression construct which was used for crystallization. (PDF 612 kb)

## Abbreviations

T7SS: type VII secretion system
ESAT-6: early secreted antigenic target of 6 kDa
ESX: ESAT six
Ecc: ESX conserved component
MBP: maltose binding protein
r.m.s.d.: root mean square deviation.

## References

[1] Hoffmann C, Leis A, Niederweis M, Plitzko JM, Engelhardt H (2008). Disclosure of the mycobacterial outer membrane: cryo-electron tomography and vitreous sections reveal the lipid bilayer structure. Proc Natl Acad Sci U S A.

[2] Pym AS, Brodin P, Brosch R, Huerre M, Cole ST (2002). Loss of RD1 contributed to the attenuation of the live tuberculosis vaccines Mycobacterium bovis BCG and Mycobacterium microti. Mol Microbiol.

[3] Guinn KM, Hickey MJ, Mathur SK, Zakel KL, Grotzke JE, Lewinsohn DM (2004). Individual RD1-region genes are required for export of ESAT-6/CFP-10 and for virulence of *Mycobacterium tuberculosis*. Mol Microbiol.

[4] Majlessi L, Brodin P, Brosch R, Rojas MJ, Khun H, Huerre M (2005). Influence of ESAT-6 secretion system 1 (RD1) of Mycobacterium tuberculosis on the interaction between mycobacteria and the host immune system. J Immunol.

[5] Champion PA, Stanley SA, Champion MM, Brown EJ, Cox JS (2006). C-terminal signal sequence promotes virulence factor secretion in Mycobacterium tuberculosis. Science.

[6] Champion PA, Champion MM, Manzanillo P, Cox JS (2009). ESX-1 secreted virulence factors are recognized by multiple cytosolic AAA ATPases in pathogenic mycobacteria. Mol Microbiol.

[7] Champion MM, Williams EA, Pinapati RS, Champion PA (2014). Correlation of phenotypic profiles using targeted proteomics identifies mycobacterial esx-1 substrates. J Proteome Res.

[8] Weerdenburg EM, Abdallah AM, Rangkuti F, Abd El Ghany M, Otto TD, Adroub SA (2015). Genome-wide transposon mutagenesis indicates that Mycobacterium marinum customizes its virulence mechanisms for survival and replication in different hosts. Infect Immun.

[9] Houben D, Demangel C, van Ingen J, Perez J, Baldeon L, Abdallah AM (2012). ESX-1-mediated translocation to the cytosol controls virulence of mycobacteria. Cell Microbiol.

[10] Coros A, Callahan B, Battaglioli E, Derbyshire KM (2008). The specialized secretory apparatus ESX-1 is essential for DNA transfer in Mycobacterium smegmatis. Mol Microbiol.

[11] Gray TA, Krywy JA, Harold J, Palumbo MJ, Derbyshire KM (2013). Distributive conjugal transfer in mycobacteria generates progeny with meiotic-like genome-wide mosaicism, allowing mapping of a mating identity locus. PLoS Biol.

[12] Serafini A, Boldrin F, Palu G, Manganelli R (2009). Characterization of a *Mycobacterium tuberculosis* ESX-3 conditional mutant: essentiality and rescue by iron and zinc. J Bacteriol.

[13] Siegrist MS, Unnikrishnan M, McConnell MJ, Borowsky M, Cheng TY, Siddiqi N (2009). Mycobacterial Esx-3 is required for mycobactin-mediated iron acquisition. Proc Natl Acad Sci U S A.

[14] Siegrist MS, Steigedal M, Ahmad R, Mehra A, Dragset MS, Schuster BM (2014). Mycobacterial Esx-3 requires multiple components for iron acquisition. mBio.

[15] Daleke MH, Cascioferro A, de Punder K, Ummels R, Abdallah AM, van der Wel N (2011). Conserved Pro-Glu (PE) and Pro-Pro-Glu (PPE) protein domains target LipY lipases of pathogenic mycobacteria to the cell surface via the ESX-5 pathway. J Biol Chem.

[16] Bottai D, Di Luca M, Majlessi L, Frigui W, Simeone R, Sayes F (2012). Disruption of the ESX-5 system of *Mycobacterium tuberculosis* causes loss of PPE protein secretion, reduction of cell wall integrity and strong attenuation. Mol Microbiol.

[17] Ates LS, Ummels R, Commandeur S, van der Weerd R, Sparrius M, Weerdenburg E (2015). Essential Role of the ESX-5 Secretion System in Outer Membrane Permeability of Pathogenic Mycobacteria. PLoS Genet.

[18] van Pittius NC G, Sampson SL, Lee H, Kim Y, van Helden PD, Warren RM (2006). Evolution and expansion of the *Mycobacterium tuberculosis* PE and PPE multigene families and their association with the duplication of the ESAT-6 (*esx*) gene cluster regions. BMC Evol Biol.

[19] Bitter W, Houben EN, Bottai D, Brodin P, Brown EJ, Cox JS (2009). Systematic genetic nomenclature for type VII secretion systems. PLoS Pathog.

[20] Houben EN, Bestebroer J, Ummels R, Wilson L, Piersma SR, Jimenez CR (2012). Composition of the type VII secretion system membrane complex. Mol Microbiol.

[21] Ramsdell TL, Huppert LA, Sysoeva TA, Fortune SM, Burton BM (2015). Linked domain architectures allow for specialization of function in the FtsK/SpoIIIE ATPases of ESX secretion systems. J Mol Biol.

[22] Rosenberg OS, Dovala D, Li X, Connolly L, Bendebury A, Finer-Moore J (2015). Substrates Control Multimerization and Activation of the Multi-Domain ATPase Motor of Type VII Secretion. Cell.

[23] Finn RD, Bateman A, Clements J, Coggill P, Eberhardt RY, Eddy SR (2014). Pfam: the protein families database. Nucleic Acids Res.

[24] McGowan S, Buckle AM, Mitchell MS, Hoopes JT, Gallagher DT, Heselpoth RD (2012). X-ray crystal structure of the streptococcal specific phage lysin PlyC. Proc Natl Acad Sci U S A.

[25] Quiocho FA, Spurlino JC, Rodseth LE (1997). Extensive features of tight oligosaccharide binding revealed in high-resolution structures of the maltodextrin transport/chemosensory receptor. Structure.

[26] Krissinel E, Henrick K (2007). Inference of macromolecular assemblies from crystalline state. J Mol Biol.

[27] Ioerger TR, O’Malley T, Liao R, Guinn KM, Hickey MJ, Mohaideen N (2013). Identification of new drug targets and resistance mechanisms in Mycobacterium tuberculosis. PLoS One.

[28] Iwashkiw JA, Vozza NF, Kinsella RL, Feldman MF (2013). Pour some sugar on it: the expanding world of bacterial protein O-linked glycosylation. Mol Microbiol.

[29] Sartain MJ, Belisle JT (2009). N-Terminal clustering of the O-glycosylation sites in the Mycobacterium tuberculosis lipoprotein SodC. Glycobiology.

[30] Hansen JE, Lund O, Tolstrup N, Gooley AA, Williams KL, Brunak S (1998). NetOglyc: prediction of mucin type O-glycosylation sites based on sequence context and surface accessibility. Glycoconj J.

[31] Zhang XL, Li DF, Fleming J, Wang LW, Zhou Y, Wang DC (2015). Core component EccB1 of the Mycobacterium tuberculosis type VII secretion system is a periplasmic ATPase. Faseb J.

[32] McLaughlin B, Chon JS, MacGurn JA, Carlsson F, Cheng TL, Cox JS (2007). A mycobacterium ESX-1-secreted virulence factor with unique requirements for export. PLoS Pathog.

[33] Tsirigos KD, Peters C, Shu N, Kall L, Elofsson A (2015). The TOPCONS web server for consensus prediction of membrane protein topology and signal peptides. Nucleic Acids Res.

[34] Drozdetskiy A, Cole C, Procter J, Barton GJ (2015). JPred4: a protein secondary structure prediction server. Nucleic Acids Res.

[35] Abendroth J, Gardberg AS, Robinson JI, Christensen JS, Staker BL, Myler PJ (2011). SAD phasing using iodide ions in a high-throughput structural genomics environment. J Struct Funct Genomics.

[36] Kabsch W (2010). Xds. Acta Crystallogr D Biol Crystallogr.

[37] Otwinowski Z, Minor W (1997). Processing of X-ray diffraction data collected in oscillation mode. Methods Enzymol.

[38] Sheldrick GM (2008). A short history of SHELX. Acta Crystallogr A.

[39] Bricogne G, Vonrhein C, Flensburg C, Schiltz M, Paciorek W (2003). Generation, representation and flow of phase information in structure determination: recent developments in and around SHARP 2.0. Acta Crystallogr D Biol Crystallogr.

[40] Cowtan K (2008). Fitting molecular fragments into electron density. Acta Crystallogr D Biol Crystallogr.

[41] Emsley P, Lohkamp B, Scott WG, Cowtan K (2010). Features and development of Coot. Acta Crystallogr D Biol Crystallogr.

[42] Murshudov GN, Skubak P, Lebedev AA, Pannu NS, Steiner RA, Nicholls RA (2011). REFMAC5 for the refinement of macromolecular crystal structures. Acta Crystallogr D Biol Crystallogr.

[43] Painter J, Merritt EA (2006). Optimal description of a protein structure in terms of multiple groups undergoing TLS motion. Acta Crystallogr D Biol Crystallogr.

[44] Arbing MA, Chan S, Harris L, Kuo E, Zhou TT, Ahn CJ (2013). Heterologous expression of mycobacterial Esx complexes in Escherichia coli for structural studies is facilitated by the use of maltose binding protein fusions. PLoS One.

[45] Gibson DG (2011). Enzymatic assembly of overlapping DNA fragments. Methods Enzymol.

[46] McCoy AJ, Grosse-Kunstleve RW, Adams PD, Winn MD, Storoni LC, Read RJ (2007). Phaser crystallographic software. J Appl Crystallogr.

[47] Kelley LA, Mezulis S, Yates CM, Wass MN, Sternberg MJ (2015). The Phyre2 web portal for protein modeling, prediction and analysis. Nat Protoc.

[48] Bricogne G, Blanc E, Brandl M, Flensburg C, Keller P, Paciorek W (2011). BUSTER version 2.10.0.

[49] Soding J, Biegert A, Lupas AN (2005). The HHpred interactive server for protein homology detection and structure prediction. Nucleic Acids Res.

[50] Moon AF, Mueller GA, Zhong X, Pedersen LC (2010). A synergistic approach to protein crystallization: combination of a fixed-arm carrier with surface entropy reduction. Protein Sci.

[51] Zhang KY, Cowtan K, Main P (1997). Combining constraints for electron-density modification. Methods Enzymol.

[52] Pettersen EF, Goddard TD, Huang CC, Couch GS, Greenblatt DM, Meng EC (2004). UCSF Chimera--a visualization system for exploratory research and analysis. J Comput Chem.

[53] Robert X, Gouet P (2014). Deciphering key features in protein structures with the new ENDscript server. Nucleic Acids Res.

[54] Baker NA, Sept D, Joseph S, Holst MJ, McCammon JA (2001). Electrostatics of nanosystems: application to microtubules and the ribosome. Proc Natl Acad Sci U S A.

[55] Li H, Robertson AD, Jensen JH (2005). Very fast empirical prediction and rationalization of protein pKa values. Proteins.

[56] Karplus PA, Diederichs K (2012). Linking crystallographic model and data quality. Science.

[57] Evans P (2006). Scaling and assessment of data quality. Acta Crystallogr D Biol Crystallogr.

[58] Chen VB, Arendall WB, Headd JJ, Keedy DA, Immormino RM, Kapral GJ (2010). MolProbity: all-atom structure validation for macromolecular crystallography. Acta Crystallogr D Biol Crystallogr.

