# Supplementary material for: Structures of EccB1 and EccD1 from the core complex of the mycobacterial ESX-1 type VII secretion system
Source: BMC Struct Biol. 2016 Feb 27;16:5. doi: 10.1186/s12900-016-0056-6 (PMC4769845; doi:10.1186/s12900-016-0056-6)
Supplement: Additional file 1: Figure S1. — Sequence alignment of EccB orthologs from M. tuberculosis H37Rv. The secondary structure elements of EccB1mt are shown at the top of the alignment. The conserved Cys residues are highlighted in blue. The vertical arrows indicate the beginning and end of the EccB1mt expression construct which was used for crystallization. (PDF 612 kb) [file 12900_2016_56_MOESM1_ESM.pdf]

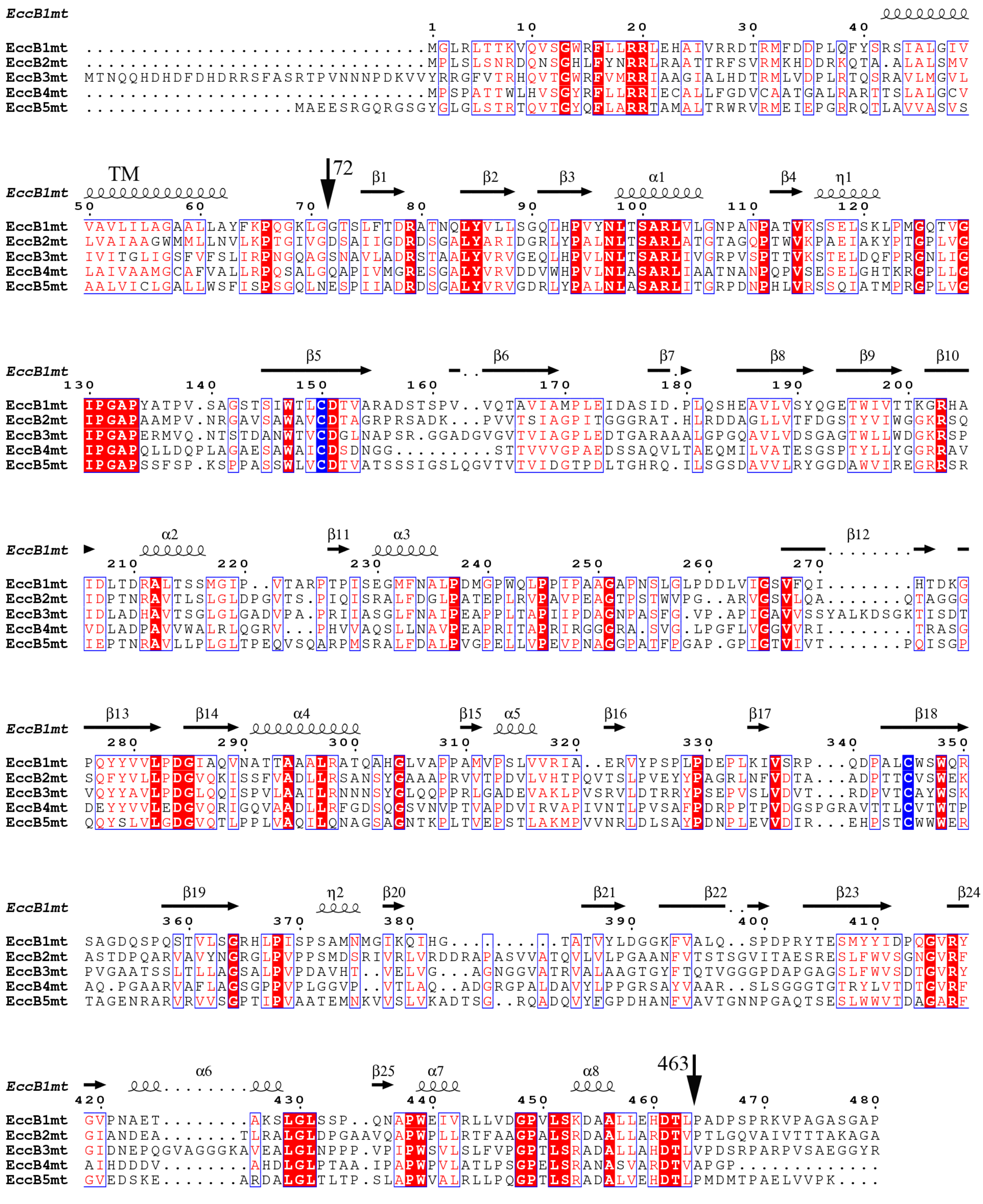

Supplementary Figure 1. Sequence alignment of EccB orthologs from *M. tuberculosis* H37Rv. The secondary structure elements of $EccB_{1mt}$ are shown at the top of the alignment. The conserved Cys residues are highlighted in blue. The vertical arrows indicate the beginning and end of the $EccB_{1mt}$ expression construct which was used for crystallization.
